# Bioterrorism: Preparing the Plastic Surgeon

**Published:** 2011-11-23

**Authors:** Karan Chopra, Alexandra Conde-Green, Matthew K. Folstein, Erin K. Knepp, Michael R. Christy, Devinder P. Singh

**Affiliations:** ^a^Division of Plastic Surgery, University of Maryland Medical Center; ^b^Section of Plastic Surgery, University of Maryland R Adams Cowley Shock Trauma Center, Baltimore, MD

## Abstract

**Introduction:** Many medical disciplines, such as emergency medicine, trauma surgery, dermatology, psychiatry, family practice, and dentistry have documented attempts at assessing the level of bioterrorism preparedness in their communities. Currently, there is neither such an assessment nor an existing review of potential bioterrorism agents as they relate to plastic surgery. Therefore, the purpose of this article is to present plastic surgeons with a review of potential bioterrorism agents. **Methods:** A review of the literature on bioterrorism agents and online resources of the Centers for Disease Control and Prevention was conducted. Category A agents were identified and specific attention was paid to the management issues that plastic surgeons might face in the event that these agents are used in an attack. **Results:** Disease entities reviewed were smallpox, anthrax, plague, viral hemorrhagic fever, tularemia, and botulism. For each agent, we presented the microbiology, pathophysiology, clinical presentation, potential for weaponization, medical management, and surgical issues related to the plastic surgeon. **Conclusion:** This article is the first attempt at addressing preparedness for bioterrorism in the plastic surgery community. Many other fields have already started a similar process. This article represents a first step in developing evidence-based consensus guidelines and recommendations for the management of biological terrorism for plastic surgeons.

The use of biological weapons for bioterrorism is a potential threat faced by many developed nations. Discerning the nature of the threat as well as an appropriate response requires awareness of the biological characteristics of these instruments of war. For the plastic surgeon, the most important means of preparing for a potential bioterrorist attack is to understand the pattern of injury of various warfare agents and the resulting reconstructive challenges. As surgical specialists with training in management of burn wounds, and cutaneous diseases requiring surgical intervention, plastic surgeons should we aware of the basic presentation and potential management of the most virulent biological warfare agents.

## HISTORY OF BIOTERRORISM

Bioterrorism involves the intentional use of organisms (ie, bacteria, viruses) or their products, such as toxins to cause death or disease. Such acts cause not only morbidity and mortality but also lead to social and political disruption. The use of biological agents as weapons dates back as early as 600 BC when militants used the remains from cadavers and animal carcasses to cause disease in soldiers of the enemy.^1^ In the 14th century, the Tatars catapulted deceased plague victims into the city, causing an epidemic amongst the inhabitants.^2^ The subsequent migration of refugees from the defeated city initiated the plague pandemic, also known as the “Black Death,” which swiftly swept through Europe and North Africa. In the 18th century, during the final battles of the French and Indian war (1754-1767), Lord Jeffrey Amherst, a commanding general of the British forces, reportedly distributed blankets that had belonged to smallpox patients with the intent of initiating outbreaks amongst American Indians. An epidemic ensued, killing more than 50% of the affected tribes.^3^

Biological warfare became more sophisticated during the 19th century with the development of Koch's postulates and modern microbiology. Less than a century later, after World War I, the United Nations officially recognized biowarfare as an international threat in the Geneva Protocol of 1925. Despite heightened awareness and international sanctions, biological weapons continued to play a role in World War II, the Persian Gulf War, and modern-day international affairs. In light of the sobering and tragic events of September 11, 2001, almost a decade ago, the threat of bioterrorism is still real and palpable; preparedness is our most potent defense.

## BIOLOGICAL WARFARE IN THE 21st CENTURY

It was not until the 2001 anthrax attacks that the United States established national public health initiatives to bolster medical preparedness. Just weeks after September 11, a string of letters sent via US mail to media organizations and political offices were found to be laced with *Bacillus anthracis* spores. A total of 22 people were injured as a result of the mailings; 11 suffered from the inhalational form of the disease, and 5 subsequently died. Thousands more were indirectly affected, including individuals working in facilities contaminated by the attacks, and their families. Because anthrax was rarely encountered in medical practice, few local or federal public health officials had experience identifying and evaluating *B. anthracis* infection. In addition, this was the first time that the Centers for Disease Control and Prevention (CDC) had been contacted to respond to outbreaks of illness occurring simultaneously in 5 major cities. This combination of lack of experience, consistency, and leadership led to medical mismanagement.^4^

## PREPAREDNESS OF THE MEDICAL COMMUNITY

The events of September 11 and the anthrax attacks exposed vulnerability to bioterrorism in the medical community.^4^ Should future attacks occur, clinicians will likely have to exercise professional judgment in the face of unfamiliar illness, poorly defined protocols, uncertain risk factors, and time constraints. Given this challenge, multiple medical disciplines including emergency medicine, trauma surgery, dermatology, psychiatry, family practice, dentistry, and nursing have all documented attempts at assessing the level of bioterrorism preparedness in their communities. Currently there is neither such an assessment nor an existing review of bioterrorism agents as they specifically relate to plastic surgery. The aim of this article is to raise awareness among plastic surgeons for such challenges by exploring the microbiology, pathophysiology, presentation, potential for weaponization, antibiotic use, medical management, vaccination, and surgical issues of specific high-priority biological agents.

## METHODS

A review of published literature on bioterrorism agents and online resources of the US CDC was conducted. We identified category A agents and paid particular attention to the management issues that plastic surgeons might face for each agent.

## RESULTS

Although any microbe could potentially be used as a weapon of bioterrorism, there are a select number of agents that pose the greatest threat because of their availability and ease of dissemination. The CDC has developed a classification system in which it assigned each potential agent a category, A through C, based on likelihood of use and risk factors. Category A agents are classified as the most effective bioweapons because they are highly virulent and contagious, require short incubation periods, and are easily produced, handled, and distributed (Table 1). Pathogens that fall into this category include the following: anthrax (*Bacillus anthracis*), botulism (*Clostridium botulinum* toxin), plague (*Yersinia pestis*), smallpox (*variola major*), tularemia (*Francisella tularensis*), and viral hemorrhagic fevers (VHFs) (filoviruses/Ebola) (Table 2).

### Smallpox

**Figure 1:**
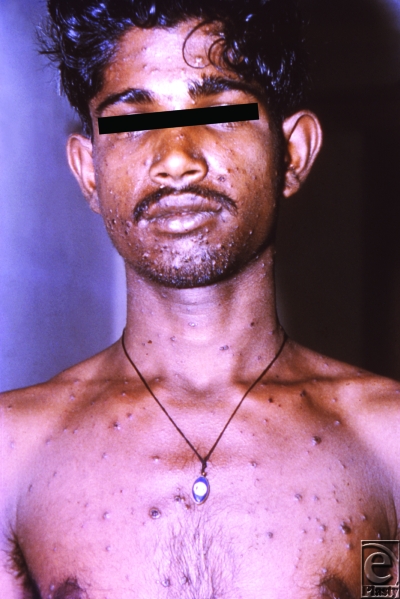
Man with small pox displaying the characteristic maculopapular rash. Source: Public Health Images Library (PHIL) id# 12165.

Smallpox is caused by the DNA virus, *Variola majora*, transmitted via inhalation of droplets. Following inoculation, the virus migrates to regional lymph nodes and multiplies. Patients typically seek medical attention 14 days after infection with complaints of high fevers, headaches, prostration, and myalgias. Then a diffuse maculopapular rash appears leaving pustules that deflate and form scabs resulting in areas of exposed dermis and subcutaneous tissue (Fig 1). Death usually occurs 6 days after the onset of the rash, with an overall mortality rate of 30% among unvaccinated persons, and a case-fatality rate for confluent smallpox as high as 62%.^5^^-^8

#### Treatment guidelines

In the event that an outbreak of smallpox would occur, treatment would include postexposure vaccination within 3 days following infection, and supportive care similar to patients with extensive skin burns.

### Anthrax

**Figure 2:**
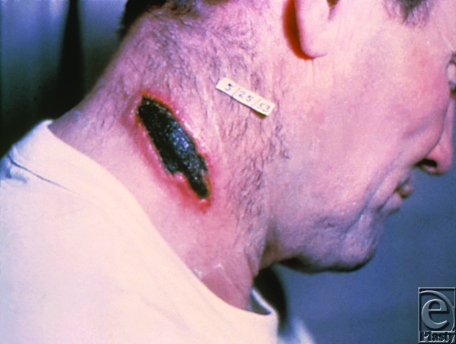
Cutaneous anthrax. Source: Public Health Images Library (PHIL) id# 1934.

Anthrax is caused by *Bacillus anthracis*, a gram-positive, encapsulated, spore-forming bacillus. It is estimated that 100 kg of powdered *B. anthracis* could cause 300,000 to 3 million deaths in a densely populated area.^9^ Infection can occur in 3 forms: inhalational, gastrointestinal, and cutaneous. Inhalational anthrax initially presents as a mild cold that progresses to respiratory failure and shock and is usually fatal.^10^ Gastrointestinal anthrax follows the consumption of contaminated meat and is characterized by gastritis and hemoptysis. When encountered via the intestinal vector, death results in 25% to 60% of cases. Cutaneous anthrax (Fig 2) may also be used as a means of warfare via direct contact of abraded skin with an inoculum from contaminated wool, hides, leather, or hair products of infected animals, or incidentally upon exposure from a failed attempt at aerosolization.

#### Treatment guidelines

First-line treatment for endemic cutaneous anthrax includes ciprofloxacin (400 mg IV [intravenously] twice daily) or doxycycline (100 mg IV twice daily) for 10 to 14 days. However, victims of bioterrorism should be treated for 60 days. Despite prompt administration of antibiotics, cutaneous lesions may still progress through the eschar phase. Debridement of the eschar is relatively contraindicated due to the risk of hematogenous spread and subsequent development of secondary pneumonic, gastrointestinal, meningeal, or septicemic anthrax.^11^ With appropriate therapy, the morality rate is 1%. An anthrax vaccine has been developed, but is currently only available to US military personnel.

### Bubonic plague

Bubonic plague caused one of the most devastating disease pandemics in history, resulting in 20 to 30 million deaths in the 14th century. *Yersinia pestis*, the causative agent, is a nonmotile, gram-negative bacillus, transmitted to humans by plague-infected fleas carried on the Norway rat species.^12^

**Figure 3:**
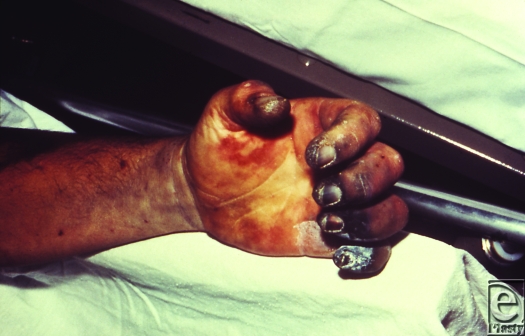
This patient presented with symptoms of plague that included gangrene of the right hand causing necrosis of the fingers. Source: Public Health Images Library (PHIL) id# 4137.

Symptoms begin 2 to 8 days after exposure, when patients start to experience prodromal symptoms. Shortly thereafter, a bubo appears in the groin, axilla, or cervical region, characterized by a swollen, erythematous, tender lymph node, 1 to 10 cm in diameter which can develop into a necrotic ulcer.^12^ A minority (13%) will develop sepsis with no bubo, a form of plague termed primary septicemic plague; leading to disseminated intravascular coagulation resulting in gangrene of acral regions such as the digits and nose (Fig 3).^13^ If left untreated, the case fatality rate for bubonic plague is 50%.^14^

#### Treatment guidelines

The recommended antibiotic for plague is parenteral streptomycin with gentamicin and doxycycline as possible alternatives. Supportive therapy includes anticoagulation and fluid resuscitation. Respiratory isolation is important for healthcare workers to prevent secondary pneumonic plague. Buboes should not be drained because they almost always recede with antibiotic therapy and incising them may cause hematogenous spread. The recommended approach is aspiration of the palpable lymph nodes, which is both diagnostic and therapeutic, providing symptomatic relief.^12^^,^^13^

### Viral hemorrhagic fever

Historically, VHF agents, such as *Arenaviridae* (Lassa fever)*, Bunyaviridae* (hemorrhagic fever with renal syndrome), *Filoviridae* (Ebola), and *Flaviviridae* (Yellow Fever and Dengue), were considered too dangerous to use as warfare weapons because they are highly contagious and unstable, putting the handler at risk for self-infection. To date, no record of VHF weaponization exists.^15^

Humans, while not a natural reservoir, may become infected through contact with infected animal reservoirs or arthropod vectors. Human-to-human transmission occurs through close contact with infected people or their bodily fluids. These viruses target the vascular bed, inducing microvascular damage and thrombocytopenic hemorrhage. Patients present acutely febrile with prostration, myalgias, fatigue, petechiae and in severe cases, frank bleeding from internal organs and mucous membranes leading to hypotension and shock. Viral hemorrhagic fever (particularly Ebola) mortality from multisystem failure is substantial, ranging from 50% to 90% within one week of exposure.^11^

#### Treatment guidelines

Treatment is largely supportive. Ribavirin has shown limited efficacy in individuals with Lassa fever or hemorrhagic fever with renal syndrome.^14^ Local wound care may be indicated for management of sequelae of microvascular damage as described earlier. Hemorrhage between fascial sheaths can sometimes precipitate an acute compartment syndrome, particularly in the forearm requiring surgical decompression.^16^

### Tularemia

**Figure 4:**
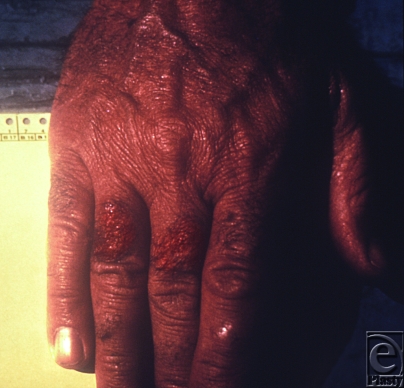
This Vermont muskrat trapper contracted tularemia, which manifested as cutaneous lesions on the dorsum of his right hand. Source: Public Health Images Library (PHIL) id# 6466. Dr. Roger A. Feldman.

Tularemia is caused by an aerobic, gram-negative coccobacillus, *Francisella tularensis*, which occurs naturally in many parts of the United States as infected rodents and ticks harbor the pathogen. Humans can become infected via insect bites, contact with infected animal carcasses, and consumption of contaminated food (Fig 4). Despite difficult handling and an unstable spore form, if tularemia were to be used as a bioweapon, the most likely method would be through aerosol spread.^11^

Once inoculated into the skin, mucous membranes, gastrointestinal tract, or lungs of a human host, these bacilli multiply intracellularly and spread to regional lymph nodes, where they disseminate throughout the body. Symptoms usually appear 5 days after exposure with a prodromal phase, a focal and intensely suppurative necrosis of the tissue which develops into granulomas (similar to patterns seen in tuberculosis and sarcoidosis).^16^ Patients with primary inhalational exposure can develop hemorrhagic inflammation of the airways, progressing to bronchopneumonia which may easily be confused with Legionnaires' disease or inhalational anthrax, so it is important to differentiate between them.^11^ Without prompt treatment, the tularemia is fatal in 80% of victims.

#### Treatment guidelines

Early antibiotic treatment with parenteral streptomycin or gentamicin can effectively control infection progression. In a mass casualty setting, oral treatment with doxycycline (14-21 days regimen) or ciprofloxacin (10 days) is preferred for those who are exposed. Precautionary isolation is not necessary as tularemia is not spread via person-to-person transmission. Skin infections presenting with a “heaped-up” ulcer may require surgical intervention, keeping in mind that incision and drainage of lymphadenopathy is contraindicated due to the risk of hematogenous spread and secondary pneumonic tularemia.^16^

### Botulism

Botulism is a paralytic neuromuscular disorder caused by the toxin of bacterium *Clostridium botulinum*, a gram-positive, spore forming, obligate anaerobe ubiquitous to soil and marine environments. There are 3 types of naturally occurring botulism—foodborne, wound, infantile—and a fourth man-made form, inhalational botulism. All forms of the disease result from the absorption of toxins into the circulation from a mucosal surface, usually intestines, lungs, and occasionally a wound. The toxin binds and irreversibly blocks the peripheral cholinergic synapses at neuromuscular junctions throughout the body causing global muscle paralysis necessitating mechanical ventilation. Botulinum toxin poses a major bioweapon threat because it is the most acutely toxic substance known with a median lethal dose as low as 1 ng/kg (IV). After the 1991 Persian Gulf War, Iraq admitted to having produced 19,000 liters of concentrated toxin, which is approximately 3 times the amount needed to kill the entire current human population through inhalation.^17^ Aerosolized dissemination of the toxin is the most likely means of biowarfare, but given the difficulty of stabilizing the agent, terrorists may prefer contaminating food products.

**Figure 5:**
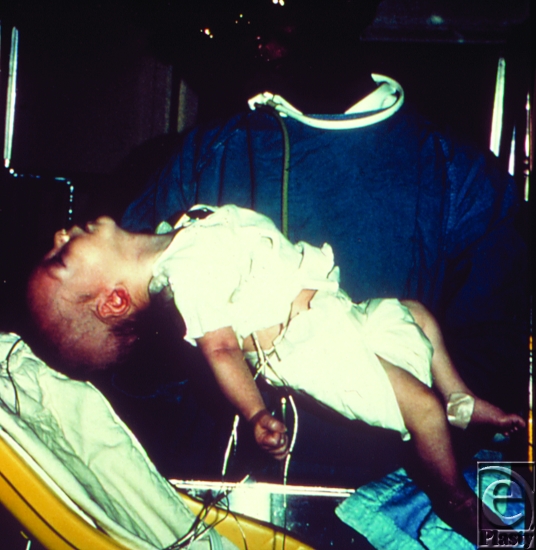
Six-week-old infant with botulism, which is evident as a marked loss of muscle tone, especially in the region of the head and neck. Source: Public Health Images Library (PHIL) id# 1935.

All forms of botulism ultimately manifest into a classic triad: (*a*) symmetric, descending flaccid paralysis with prominent bulbar palsies (with diplopia, ptosis, dysarthria, dysphonia, and dysphagia), (*b*) afebrile patient with (*c*) clear sensorium. Infants with botulism present with lethargy, poor feeding, poor muscle tone, and a weak cry (Fig 5). The neurologic symptoms from foodborne botulism, the most common form, may be preceded by abdominal cramps, vomiting, or diarrhea.^18^ Rapidity of onset and severity of botulism depend on the rate and amount of toxin absorption^19^ and the route of administration.

#### Treatment guidelines

Treatment consists of supportive care and immunization with equine antitoxin. Respiratory failure secondary to paralysis can persist for weeks to months, requiring extended mechanical ventilation and intravenous fluid resuscitation. If diagnosed early, administration of a passive neutralizing antibody can minimize subsequent nerve damage and severity, which will prevent symptom progression. If wound botulism is suspected, wide debridement to remove the source of the toxin-producing bacteria is indicated. Because of early recognition and improved care, the mortality rate amongst botulism patients has fallen from about 50% to 8% over the past 50 years.

## DISCUSSION

As demonstrated by the anthrax letters and the aftermath of the World Trade Center attack, biological terrorism is a real and present threat. The highly potent “Class A” agents discussed earlier can inflict serious illnesses on thousands of victims in a relatively short period of time. Even a small-scale attack can create an enormous psychological impact when the medical community is ambushed with an unfamiliar threat, and unpreparedness can certainly result in chaos. In 2001, for example, the anthrax attacks prompted the CDC, local public health agencies, and community physicians to confront an array of scientific uncertainties. They had limited experience in dealing with *B. anthracis* disease, and CDC officials reported at the time: “We lacked scientific data to address issues. We could not inform public health decision making regarding issues such as exposure, isolated cases, letters in transit, and cross-contamination.”^4^ The usual systematic, step-by-step approach of the CDC for investigating disease outbreaks was simply not feasible in the context of a high-profile multifocal attack that potentially placed thousands at risk. Furthermore, most local health practitioners had never seen a single case of inhalational anthrax. They were on the “front lines” without the experience, guidance, or support to contain the threat and effectively treat the victims.

This episode also served as a tremendous impetus to bolster bioterrorism education amongst the medical community. Because this issue globally involves all disciplines, it is equally important that plastic surgeons be primed with treatment guidelines if consulted in the event of a future attack. In our opinion based on our experience at a large urban hospital, most plastic surgeons have little, if any, experience with “Class A” infectious agents. At a minimum, we should be familiar with the basic presentation of these bioterrorism agents. Because these attacks tend to affect a large number of victims simultaneously, the medical community is likely to be overwhelmed and require the assistance of physicians such as plastic surgeons who may not otherwise be thought of as primary responders to most of these disease entities. Furthermore, when a large number of patients present with manifestations of disease caused by biological agents, they may occupy a large number of patient beds in the emergency departments, and hospitals where the attack has occurred. The ability to correctly identify these manifestations at an early stage is the only way physicians can manage and treat these patients. Unless these cases are correctly identified, these patients may occupy beds in the emergency department indefinitely and compromise care for patients with other acute illnesses that require rapid workup. On the contrary, patients who are dismissed from the emergency department with an incorrect diagnosis may spread the disease throughout the community or even die from the disease. The first challenge this article addresses is providing the information to discern index cases rapidly and correctly. As described earlier, patients subjected to bioterrorist agents can present with neurologic symptoms (eg, botulism), respiratory symptoms (eg, plague pneumonia, inhalation anthrax, tularemic pneumonia), gastrointestinal illnesses, or hemolytic (eg, African hemorrhagic fevers) symptoms. In addition, it establishes basic guidelines for surgical management options in regard to each pathogen, because many present with soft tissue and osseous manifestations.

## CONCLUSION

A brief glance at peer-reviewed resources indicates that many other fields have already started a process of addressing preparedness for bioterrorism in their medical communities. A pilot assessment survey to determine the current level of preparedness is underway. This article represents a first step in developing evidence-based consensus guidelines and recommendations for the management of biological terrorism for plastic surgeons.

## Figures and Tables

**Table 1:** Characteristics of an effective bioweapon

Highly virulent
Highly contagious
Short incubation period
Susceptibility of target population
Easily dispersed
Easily produced
Easily handled

**Table 2:** Summary table of biological warfare agents

Agent	Mortality	Potential Plastic Surgery Consultation
Smallpox	30% without pre- or postexposure vaccination	• Lesions may become confluent with resultant skin slough. Potential for burn-like care and resuscitation
		• Bacterial super-infection of skin may occur
		• Vaccine complications include skin necrosis at inoculation site (ie, Vaccinia necrosum)
Anthrax	20% in the untreated cutaneous form	• Even with prompt antibiotic therapy, cutaneous lesions progress through eschar phase
		• Debridement relatively contraindicated due to risk of hematogenous spread and secondary pneumonic anthrax
Plague	50% in the untreated group	• Erythematous, eroded, crusting, necrotic ulcer at primary inoculation site
		• Incision and drainage of lymphadenopathy (buboes) is contraindicated due to the risk of hematogenous spread and subsequent, secondary pneumonic plague
		• Respiratory isolation important for healthcare workers to prevent secondary pneumonic plague
VHF (ie, Ebola)	50%-90% within 1 wk	• Mucosal and/or cutaneous ecchymoses common, can be associated with overlying skin slough
		• Rule out acute compartment syndrome with extremity involvement
Tularemia	80% in untreated inhalational form	• “Heaped-up” ulcer at primary inoculation site
		• Incision and drainage of lymphadenopathy (“plague-like” buboes) is contraindicated due to the risk of hematogenous spread and secondary pneumonic tularemia
Botulism	60% in the untreated group	• Terrorist attack likely to be in aerosolized form, causing inhalational botulism. Requiring respiratory support for flaccid paralysis
		• If wound botulism is suspected as cause of flaccid paralysis, wide debridement is indicated

VHF indicates viral hemorrhagic fever.

## References

[1] Riedel S (2004). Biological warfare and bioterrorism: a historical review. Proc (Bayl Univ Med Cent).

[2] Derbes VJ (1996). De Mussis and the great plague of 1348: a forgotten episode of bacteriological war. JAMA.

[3] Stearn EW, Stearn AE (1945). The Effect of Smallpox on the Destiny of the Amerindian.

[4] Gursky E, Inglesby TV, O'Toole T (2003). Anthrax 2001: observations on the medical and public health response. Biosecur Bioterror.

[5] Henderson DA, Inglesby TV, Bartlett JC (1999). Smallpox as a biological weapon: medical and public health management. JAMA.

[6] Fenner F, Wittek R, Dumbell KR (1988). The Orthopoxviruses.

[7] Wehrle PF, Posch J, Richter KH, Henderson DA (1970). An airborne outbreak of smallpox in a German hospital and its significance with respect to other recent outbreaks in Europe. Bull World Health Organ.

[8] Atkinson W, Wolfe S, Hamborsky J, McIntyre L, Centers for Disease Control and Prevention (2009). Epidemiology and Prevention of Vaccine-Preventable Diseases. Smallpox.

[9] Centers for Disease Control and Prevention (1999). Emerging Infectious Diseases.

[10] Sidell FR, Patrick WC, Dashiell TR (1998). Jane's Chem-Bio Handbook.

[11] Cunha B (2002). Anthrax, tularemia, plague, ebola or smallpox as agents of bioterrorism: recognition in the emergency room. Clin Microbiol Infect.

[12] Butler T, Mandell GL, Bennett JE, Dolin R (1995). Yersinia species (including plague). Principles and Practice of Infectious Diseases.

[13] Centers for Disease Control and Prevention (1997). Fatal human plague. MMWR Morb Mortal Wkly Rep.

[14] Dennis DT, Inglesby TV, Henderson DA (2001). Consensus Statement: Tularemia as a Biological Weapon: Medical and Public Health Management. JAMA.

[15] (2006). Weapons of Mass Casualties and Terrorism Response Handbook.

[16] Moght A, Alavi-Naini R, Azimi H (2005). Compartment syndrome: an unusual course for a rare disease. Am J Trop Med Hyg.

[17] Zilinskas RA (1997). Iraq's biological weapons: the past as future?. JAMA.

[18] Hughes JM, Blumenthal JR, Merson MH, Lombard GL, Dowell VR, Gangarosa EJ (1981). Clinical features of types A and B food-borne botulism. Ann Intern Med.

[19] Koenig MG, Drutz D, Mushlin AI, Schaffer W, Rogers DE (1967). Type B botulism in man. Am J Med.

